# What the earliest evidence for life tells us about the early evolution of the biosphere

**DOI:** 10.1098/rstb.2024.0106

**Published:** 2025-08-07

**Authors:** Frances Westall

**Affiliations:** ^1^ Centre de Biophysique Moléculaire, CNRS, Orléans, France

**Keywords:** early life, chemotrophy, phototrophy

## Abstract

Life emerged on Earth in an ultramafic world under anaerobic conditions and conditioned by particular environmental characteristics for which no record remains. Molecular clocks suggest that the Last Universal Common Ancestor, LUCA, lived in a well-established biome towards the end of the Hadean, between 4.33 and 4.09 Ga. They also suggest that the last bacterial common ancestor (LBCA) and the last archaeal common ancestor (LACA) may have diverged by the Palaeoarchaean, i.e. earlier than suggested by phylogenetic dating. Moreover, various geochemical and isotopic proxies for specific microbial metabolisms have been used to date the appearance of certain metabolic pathways in geological time. According to some molecular clocks, oxygenic photosynthesis arose in the Palaeoarchaean (3.5–3.2 Ga) and some geochemical studies point to oxygenic photosynthesis already in Eoarchaean times. The bulk of the geological evidence, however, indicates its appearance in the Mesoarchaean (3.2–2.8 Ga). This contribution explores the geological and palaeontological evidence for these interpretations and cautions the need to take into account other, abiotic influences on the proxy signatures, as well as the importance of basing interpretations of biogenicity on a complementary suite of proxies to ensure correct elucidations.

This article is part of the discussion meeting issue ‘Chance and purpose in the evolution of biospheres’.

## 1. Introduction

**Figure 1.**
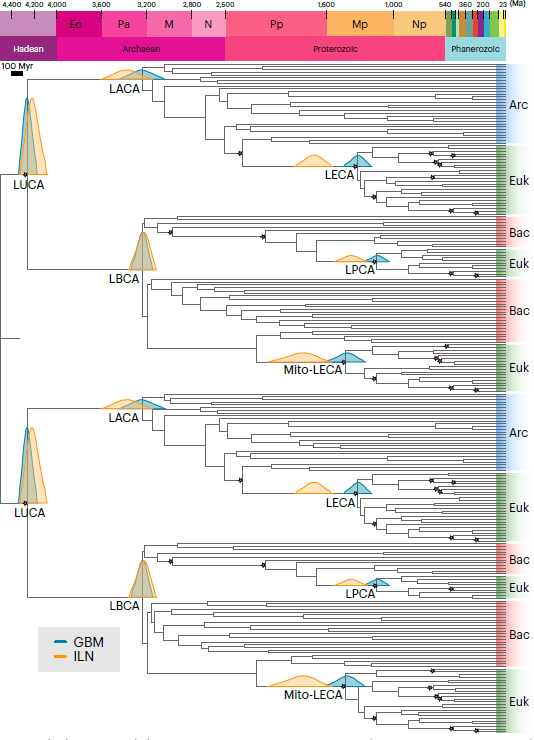
Time tree constructed by Moody *et al*. [13] which places LUCA (and its ecosystem) at about 4.2 Ga, showing also the estimated timings of appearance of the last universal, archaeal, bacterial and eukaryotic common ancestors (LUCA, LACA, LBCA and LECA, respectively); the last common ancestor of the mitochondrial lineage (Mito-LECA); and the last plastid-bearing common ancestor (LPCA). Arc, Archaea; Bac, Bacteria; Euk, Eukarya. The earliest parts of this figure are bracketed by the date of the Moon-forming impact (a date of 4.52 Ga is used by Moody *et al*. [13], but see discussion below), and the oldest known fossil evidence for cellular life. Likewise, the latter is grounded in the Betts *et al*. [14] paper, which refers to enigmatic structures in the 3.35 Ga Strelley Pool Chert, Panorama Formation, Pilbara [15]. We note that there are, in fact, older cellular fossils in the 3.45 Ga Kitty’s Gap Chert, Pilbara [9,16].

According to the Nobel Laureate, Christian de Duve, the appearance of life as we know it should be a natural consequence of the evolution of chemistry into biology—in certain environmental conditions that may be common in the Universe [1]. Unfortunately, on Earth, these specific environmental conditions are difficult to access because of impact and tectonic recycling of the Hadean crust (see electronic supplementary material for information on the conditions and subsequent availability of electron donors and acceptors for microbial metabolism). There are tenuous signs of life in rocks 3.8−3.7 billion years old [2–4], and abundant signs of life in younger (as of 3.5 Ga), more well-preserved rocks (e.g. [5–12]), all associated with an anoxic environment (at least until about 3.0 Ga). However, the evolution of life seems to have been rapid because, already by 3.5 Ga, life had diversified and comprised phototrophs as well as chemotrophs and, by 3.0 Ga, there is circumstantial evidence of oxygenic photosynthesis. Moreover, various molecular clocks suggest a very early appearance of life on Earth with the latest estimates dating the Last Universal Common Ancestor, LUCA, between 4.33 and 4.09 Ga (mean approx. 4.2 Ga) [13] (figure 1). LUCA was probably a prokaryote-grade organism using an anaerobic acetogenic metabolism for growth and carbon fixation that lived in an established ecosystem and had a genome similar in size to that of modern prokaryotes (*ca* 2.5 Mb) (*op. cit*.). Furthermore, some molecular clocks suggest that oxygenic phototrophy appeared during the Palaeoarchaean [17–19], although the environmental evidence strongly indicates anoxic conditions, both globally and locally. Today, the dominant oxygenic phototrophs are cyanobacteria that, according to Sánchez-Baracaldo *et al*. [19], appeared during the Mesoarchaean and before the Great Oxidation Event (GOE). The Palaeo-Mesoarchaean was therefore a critical period when fundamental changes occurred in microbial metabolisms, changes that are difficult to discern in the rock record. These are the changes that permitted oxygenic photosynthesizers to eventually dominate the Earth’s surface during the Proterozoic and heralded the emergence of eukaryotes [20].

In this paper, I will provide a brief overview of the life forms that flourished in the different habitable niches that were available on the early Earth, concentrating on evidence related to specific metabolisms. The importance of using a variety of types of biosignatures for reliable interpretation of biogenicity is highlighted and discussed in detail in the electronic supplementary section. Given the significance of the evolution of oxygenic phototrophy for the subsequent evolution of life, I will highlight in particular the evidence for phototrophs. At the same time, I will caution that the various proxies used for interpreting microbial metabolisms may have been compromised by the high flux of abiotic organic matter on the early Earth, as well as endogenous terrestrial sources, an aspect hitherto not acknowledged.

## 2. Early life on an anaerobic, ultramafic and volcanically active planet

Early life emerged on a waterworld under environmental conditions completely different to those of today [20–22]. Barring any hypotheses of panspermia, the dispersion of life through space [23,24], the emergence of life was entirely conditioned by the environmental context of the early Earth. Pertinent for this discussion is a recent re-evaluation of the age of the Moon-forming impact, dated at 4.36 Ga [25] as opposed to the earlier date of 4.52 [26] or 4.51 Ga [27]. The earlier dates likely measured the formation ages of solar constituents of the embryo Moon rather than the collision of two planets and their consequent melting and reconsolidation [25]. This places an interesting constraint on the timing of the emergence of life (later rather than earlier), and the timing of LUCA, proposed on the basis of molecular clock analysis to have lived between 4.33 and 4.09 Ga [13]. If the Moon-forming impact occurred around 4.35 Ga and the oceans were ‘habitable’ in the sense of the emergence of life (cf. [21,28]) as of 4.34 Ga, life could have emerged very soon after. Many scenarios have been proposed for the emergence of life that are not the subject of this work and have recently been reviewed [21,22,28,29].

Also of interest for this discussion is the fact that the contribution of organic components of extraterrestrial and endogenous origin (i.e. formed in the atmosphere and in the crust) to the early Earth was significant [30–35]) and may have impacted morphological, elemental and isotopic traces of early life to the extent that metabolic processes interpreted from isolated measurements are equivocal (see discussion in electronic supplementary material). Based on what we understand of microbial metabolisms today, all the potential electron donors and acceptors for microbial cellular activity were present on the early Earth, except for large amounts of free oxygen and other components that required oxygenated conditions in order to be available or assimilable, such as sulfate, nitrate, manganese oxide and molybdenum.

**Figure 2.**
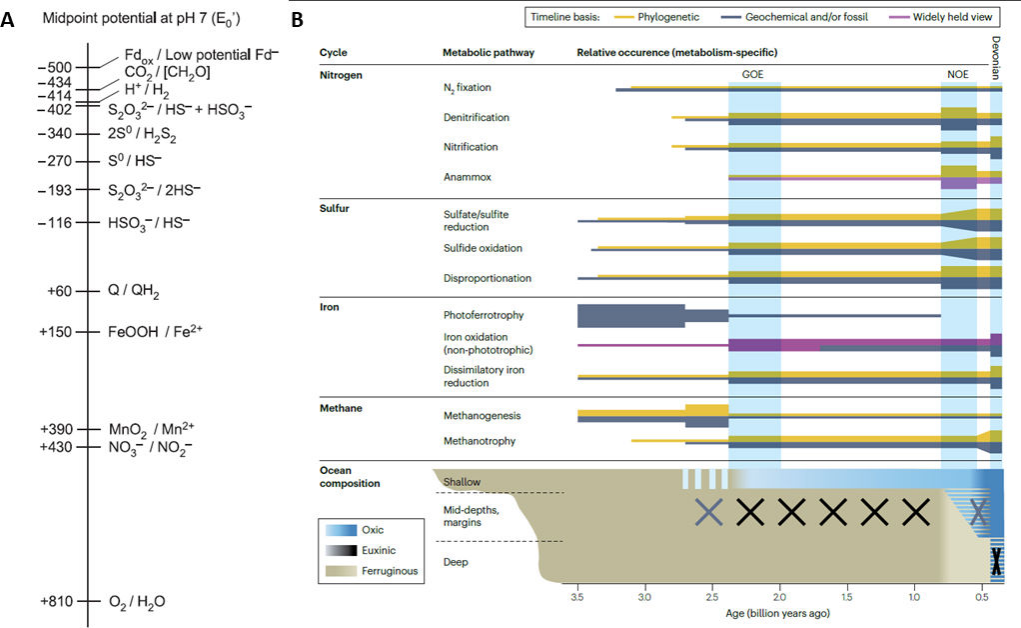
Energy couples available for life and microbial metabolisms through time. (A) Midpoint potentials of some redox couples relevant to this article illustrating the enormous advantage of oxygenic photosynthesis [36]. Fd, ferredoxin; Q, quinone. (B) Co-evolution of oceans and microbial pathways on Earth through time [37].

Figure 2A illustrates the amount of energy available from known redox couples, with the lowest energy fluxes coming from early chemotrophic metabolisms, i*.*e*.* the redox couples H_2_/H^+^; HS^–^ or HSO_3_^–^/S_2_O_3_^2–^ [36]. At the other end of the spectrum, it is the oxidation of H_2_O to O_2_ that produces the most energy. In parallel, (figure 2B) illustrates the evidence for the different metabolisms based on nitrogen, sulfur, iron and methane back through time using phylogenomic estimates and evidence from the rock record (after [37]).

On the subject of oxygen, it should be kept in mind that there were a number of abiotic means of producing free oxygen at the surface of the early Earth. The flux of UV was much higher compared to today [38] and would have affected the mostly shallow water environments in which the majority of the biosignatures occur. In these environments, photolysis of the seawater would have produced small amounts of free oxygen. Another method is the photolytic dissociation of high pressure water vapour as it exits shallow hydrothermal vents that would have been common on the early Earth [39]. Koppenol & Sies [40] and Haqq-Misra *et al*. [41] explored other processes that could have produced reactive oxygen species (e.g. H_2_O_2_) on the early Earth via ionizing radiation, reactions in the atmosphere involving CO_2_, H_2_O, lightening and UV radiation. Thus, small amounts of oxygen would have been available for some of the redox reactions listed in figure 2A.

In the following, I will examine the evidence for the appearance of specific metabolisms in the rock record. I will not list all the evidence for life from the Hadean to the Mesoarchaean; this has already been done by others [20,42,43]. Note that many of the oldest biosignatures are subject to misinterpretation owing to similarity with features, morphological or organic, of abiotic origin, either extraterrestrial, atmospheric or crustal. The problems of syngenicity and biogenicity are explained at length in the supplementary data but I here present a brief overview of the constraints regarding interpretations of biogenicity and metabolic pathways.

### (a) Constraints on the interpretation of biogenicity

Interpretations of microbial life and its characteristics in the rock record (biogenicity) in the early history of the Earth, i.e. the Archaean, 4.0−2.5 Ga, and even the latter part of the Hadean (4.5−4.0 Ga), are predicated on the existence of biological phenomena that can survive encapsulation in sediments and preservation over geological time and throughout geological processes, such as metamorphism and tectonism. Detailed explanations of the preservation process (taphonomy) are covered by Brasier *et al*. [44], Westall & Cavalazzi [45], Westall & Hickman-Lewis [46], Manning-Berg *et al*. [47] and only a brief overview is given here. In the first place, not all microorganisms are ‘fossilizable’, i.e. can be encapsulated in a mineral cement [48], and most are degraded to a lesser or greater extent before being entombed in a mineral matrix. The organic components of a cell or community rapidly degrade, are re-utilized by heterotrophic microorganisms and, especially in oxidizing conditions, may be completely oxidized. The sediments are lithified and subjected to mechanical and metamorphic degradation throughout geological time. Thus, only a part of the microbial signatures at any given place and time is preserved, therefore the record is patchy. Especially with respect to the oldest part of the rock record, the Hadean/Archaean, interpretation of traces of life need to be subjected to rigorous scrutiny and based on a variety of evidence, for example, morphological evidence, the isotopic ratios of C, S, N and Fe (as well as others that have been used), the compositional and structural characteristics of preserved organic matter, and indirect indications, such as biominerals or bioconstructions (see electronic supplementary material for details; also [45,49,50]). The use of a variety of different types of biosignatures to augment the reliability of identification is necessitated by the fact that many abiotic structures of mineral and/or organic origin may resemble biological features (e.g. [51,52]).

Interpretations of metabolic pathways from the rock record have been based on morphological aspects, such as structures resembling cyanobacteria (and therefore automatically oxygenic phototrophy, e.g. [53], but see [54]), or bioconstructions including stromatolites [6,55] or microbially induced sedimentary structures (MISS, e.g. [56]), both the result of phototrophic microorganisms. However, metabolic interpretations are primarily based on carbon [57], sulfur [58], nitrogen [59] and iron isotopes [60]. Nevertheless, there may be a potential complication regarding use of these isotopic ratios to interpret metabolism. Garcia *et al*. [61] question the strange similarity of the carbon isotope signature of the enzyme RuBisCO throughout geological time (cf. [37,57]), despite the fact that it should have changed concomitant with the significant appearance of oxygen in the environment, i.e. the Great Oxidation Event (GOE) at about 2.4–2.3 Ga, and its subsequent behaviour during the Proterozoic to a final increase in the Cryogenian (approx. 800 Ma). One of the major factors that might have influenced the isotopic ratios of elements in biogenic organic matter on the early Earth was contamination from abiotic sources or organic carbon. These included extraterrestrial flux in carbonaceous chondrites [62,63], the formation of organic molecules in the primitive atmosphere [30–35] or the formation of molecules in the Earth’s crust [64]. Of these, the greatest influence came from extraterrestrial organics whose elemental isotopic fractionations overlap with those of microbial organisms (discussed extensively in the electronic supplementary material).

Various proxies for the presence of oxygen in the environment (and, therefore, the implication of oxygenic photosynthesizers) include uranium [65,66], molybdenum or rhenium that are more readily mobilized in the oxidized form, for which a certain amount of oxygen in the environment is necessary, and negative Ce anomalies [67–70]. The presence of abundant sulfite or nitrate in the oceans (as of the GOE) also required oxygen [37]. However, very small amounts of uranium, molybdenum and sulfate were present already in sediments dating from the Eoarchaean (4.0−3.6 Ga), well before the GOE [65,67,71]. As noted above, other means of producing local oxygen through photolysis of H_2_O existed on the early Earth, especially in the shallow water regions from which the majority of Eo-Palaeoarchaean sediments originate [21,40,41], that could account for the ‘faint whiffs of oxygen’ (cf. [67]).

A final constraint concerns mixed signatures. For example, one simplistic distinction is the classification of microorganisms into ‘chemotrophs’ (organisms obtaining carbon from CO_2_ and their energy from oxidation of inorganic substrates (e.g. H_2_, chemolithotrophs) or from pre-existing carbon (chemoorganotrophs)) or phototrophs. Phototrophic organisms obtain their energy from sunlight. However, colonies of microorganisms display many variations in terms of energy sources, depending upon availability on a microscale in the environment. An example of this is a stratified mat produced by oxygenic phototrophs whose different underlying layers are oxidized by aerobic heterotrophs, anoxygenic phototrophs, various anoxygenic heterotrophs, sulfate reducers and, finally methanogens, with increasing depth in the mat [72]. Thus, any bulk analyses of preserved organic matter in rocks, or even analyses on a millimetre scale, may reflect a multitude of metabolic strategies (and this apart from the possibility of the admixture from abiotic sources, especially on the early Earth (cf. [32]).

### (b) Hadean

Given the lack of Hadean rocks (the Acasta gneiss is 4.02 Gyr [73] but contains no evidence of life), the evidence of life in the Hadean is rare and contentious. Graphitic inclusions in a 4.1 Gyr old inherited zircon crystal from Jack Hills in Western Australia have carbon isotope signatures of –24‰ that are consistent with biological fractionation [74], but this value is also consistent with isotope signatures resulting from crustal or extraterrestrial processes, although the authors discard these possibilities. Moreover, as noted above, Garcia *et al*. [61] question the reliability of carbon isotope signatures based on modern metabolic pathways throughout geological time as a proxy for microbial enzymatic reactions using RuBiSCO.

Possibly dating from the Hadean (4.3 Ga), but most likely of Eoarchaean age (3.7 Ga) is the Nuvvuagittuq belt in Quebec [4,75], which has a 2.5 Gyr hydrothermal overprint [76]. A combination of morphological and carbon and sulfur isotope signatures in chemical sediments, possibly a banded iron formation (BIF), was interpreted to suggest the presence of life. Filamentous structures outlined by hematite were interpreted as microfossils, the remains of iron-oxidizing bacteria, while the δ ^34^S enrichment was explained by microbially mediated elemental sulfur reduction, or disproportionation of sulfite (SO_3_). However, abiotic processes could be argued to explain both the morphological (e.g. [20,51,77]) and isotopic signatures (see the electronic supplementary material). Moreover, iron-oxidising bacteria are generally oxygenic, although anaerobic phototrophs may also use this metabolism. This would imply the early appearance, at a minimum, of anaerobic photosynthesizers. If the deposits are indeed Hadean in age and the evidence for life confirmed, this would imply an early start to life, before 4.3 Ga, and very rapid evolution given the likelihood of a late Moon-forming impact at about 4.36 Ga [25]. Moody *et al*. [13] suggest that LUCA lived in a 4.2 Gyr (range 4.1−4.3) biome; this would place the Nuvvuaggituq microbes pre-LUCA. On the other hand, if they are biogenic and of Eoarchaean in age, this would concur better with the phylogenetic evolutionary tree and what is understood from the record of biosignatures.

### (c) Eoarchaean

While evidence for life in the Hadean is particularly chimerical, Eoarchaean rocks of 3.7−3.8 Gyr from the Isua Supracrustal Belt are reported to contain a number of different biosignatures. The isotopic signature of carbon in these rocks was interpreted as representing oxygenic photosynthesis. However, the reported signatures (−16.1 from Schidlowski [78]; −19.07 to −19.11‰ from Rosing [2]; −25.6‰ from Rosing & Frei [65]) also overlap with those of anoxygenic photosynthesizers, as well as abiotic carbon (see electronic supplementary material). Moreover, Westall & Folk [79] noted the presence of recent and recently fossilised phototrophic endoliths in these sediments that could confuse measurements.

**Figure 3.**
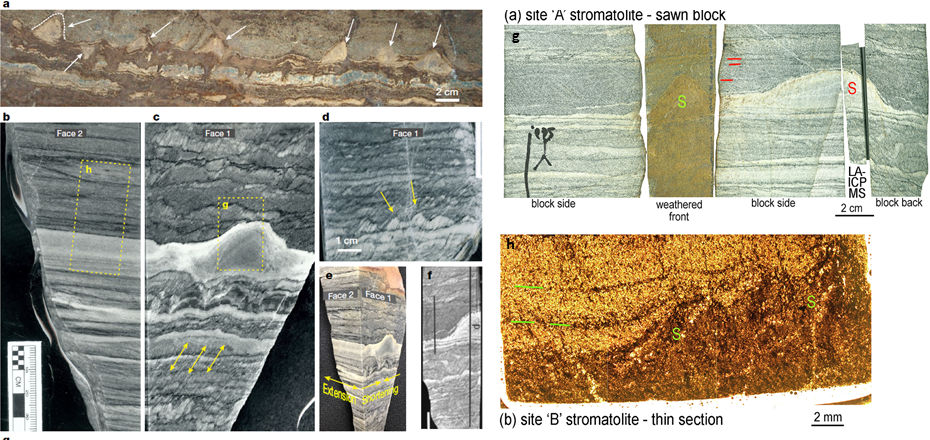
Purported stromatolites from 3.7 to 3.8 Ga Isua Supracrustal Belt. (a) Field view of the interpreted conical stromatolitic structures from Isua. (b–f) Cut faces of rock sections illustrating strain features. (a–f from [82]). (g,h) Cut rock faces across the stromatolitic structures with detail in (h) illustrating interpreted biofilm onlap onto vertical structures (g, h from [81]).

Nutman *et al*. [80,81] investigated domical structures in a recently exposed rock surface that exhibited morphological characteristics similar to those described from the 3.4 Gyr Strelley Pool Chert in the Pilbara [6,55] that they interpreted as stromatolites (figure 3). (Stromatolites were defined by Krumbein [83] as ‘laminated rocks, the origin of which can clearly be related to the activity of microbial communities, which by their morphology, physiology and arrangement in space and time interact with the physical and chemical environment to produce a laminated pattern which is retained in the final rock structure’.) However, problems related to the highly strained and metamorphosic nature of the rocks complicate interpretations (cf. [82]).

Building upon the hypothesis of oxygenic plankton phototrophs existing at the time the Isua sediments were deposited, and using Pb isotopic compositions to determine a high primary U/Th composition of the early seawater, Rosing & Frei [65] proposed that strongly oxidizing conditions must have existed in order to transport the uranium. However, evidence for strongly oxidizing conditions during Isua times have not been supported, although evidence of possible anoxygenic photosynthesis is provided by a study using iron isotopes in a BIF from the Isua supracrustal rocks by Czaja *et al*. [60]. Anoxygenic photosynthetic Fe^2+^ oxidation (e.g. [84]) or UV photooxidation [85] would be the only means of oxidising Fe of hydrothermal origin in Eoarchaean times. The δ^56^Fe values ranging from +0.4‰ to +1.1‰ measured by Czaja *et al*. [60] in the magnetite-rich layers of the Isua BIF are slightly higher than those measured in carbonaceous chondrites (electronic supplementary material, [86]). Non-phototrophic iron oxidation requires oxygen and there is no geochemical evidence for significant amounts of this before about 3.0 Ga. Fe^3+^ produced either by Fe^2+^ oxidation by chemolithoautotrophs or via anoxygenic photosynthesis (photoferrotrophy) can be used in dissimilatory iron reduction (DIR) (i.e. anaerobic microbial respiration of biomass coupled to Fe^3+^ reduction). The result of DIR is the release of Fe^2+^ into the environment causing the precipitation of Fe^2+^-bearing minerals, such as sulfides, phosphates or carbonates, thus contributing to the formation of the BIFs. (Note, however, that Martin *et al*. [36] suggest that photoferrotrophy may be a recent adaptation of chlorophototrophs due to horizontal transfer of cytochromes that can oxidize Fe^2+^ using O_2_ as the terminal electron acceptor.)

The Isua Supracrustal rocks provide a number of indications that life may have been present on the Eoarchaean Earth. Although each investigation mentioned above concentrated on a single type of biosignature, the combined evidence of possible morphology, carbon and iron isotopes, and elemental composition (including nitrogen, cf. [87]) of remnant carbonaceous matter is suggestive of the presence of life, although not definitive because of the various possible alternative abiotic sources for each potential biosignature. Particularly, in rocks that have been significantly altered, more lines of evidence would better support the interpretations. Nevertheless, given the abundance of manifestations indicating the widespread distribution of life in the Palaeoarchaean as of 3.48 Ga, and evidence of its relative diversification, it is strongly likely that life was present in Eoarchaean times.

### (d) Palaeoarchaean life

As reviewed by Westall & Xiao [20], there is abundant evidence for widespread life in Palaeoarchaean sediments owing to the better preservation of the rocks. As with the comments relating to potential biosignatures in Hadean and Eoarchaean materials, the fact that a number of abiotic phenomena can imitate microbial signatures means that much caution needs to be taken in evaluating the signatures and in their interpretation, as discussed in the electronic supplementary material. I will use only a few studies of Palaeoarchaean biosignatures to illustrate the possible appearance of key metabolisms.

#### (i) Chemotrophs

While the proposed Eoarchaean biosignatures concerned specifically photosynthesis, occurrences of chemotrophic biosignatures in the rock record are rare. A minimal amount of energy is needed for cell growth, as well as simple cell maintenance, and the steady-state population size depends on new cell growth balanced by cell removal, all controlled by energy flux [88]. In terms of energy availability, chemosynthesis produces a much lower supply of energy than photosynthesis and is believed to have appeared earlier than photosynthesis [36].

Ueno *et al*. [8,89] used the carbon isotope values of up to –56‰ in organic matter (kerogen) preserved in hydrothermal veins from the 3.48 Gyr Dresser Formation in the Pilbara Greenstone Belt, Western Australia to interpret the presence of anaerobic chemoautotrophs, such as methanogens. Their rationale was that the hydrothermal veins formed in the deep ocean and were therefore inconsistent with the possibility of phototrophic life. While the nitrogen isotopic ratio was widely variable, averaging −0.6‰, consistent with a N-fixating metabolism, the large carbon fractionation could have been produced by a reductive acetyl-CoA pathway, such as that used by methanogens or acetogens dependent upon H_2_ oxidation. Ueno *et al*. [89] did consider the possibility that the organic matter could have been produced by abiotic crustal processes, such as Fischer–Tropsch synthesis (cf. [90]), but concluded that the necessary catalysts for such a reaction, such as magnetite, hematite and Fe–Ni alloy, would not have been stable during the formation of the chert vein. In a later study of the hydrothermal veins using quadruple sulfur isotope analysis of pyrite grains, Ueno *et al*. [58] interpreted microbial activity using sulfate reduction, with the sulfate possibly derived from abiological or microbial disproportionation of magmatic SO_2_ or elemental sulfur. The interpretations of Ueno *et al.* [8,58,89] were predicated upon their deep-sea interpretation of the field context for the hydrothermal veins, at least for the methanogenic origin of the carbon isotopes. Subsequent field studies, however, have shown that the Dresser Formation did not form in the deep sea, but rather in very shallow water and possibly associated with subaerial volcanic calderas [11,91,92].

Phototrophic microbial mats in the form of MISS [11], stromatolites [93] and microbial mats associated with hot spring deposits [92] occur in the Dresser Formation. Moreover, Philippot *et al*. [94] concluded that microorganisms on the seafloor at the time of deposition of the Dresser sediments used disproportionation of elemental sulfur, rather than sulfate reduction. However, as documented in the supplementary data, the strong isotopic fractionations of carbon measured by Ueno *et al*. [8,89] also overlap with those measured in compounds produced in hydrothermal carbonaceous matter [90]. The fact is that hydrothermal fluids passing through layers of sediment containing carbonaceous matter of biogenic and/or abiotic origin could have been entrained into the Dresser Formation veins.

Another study of carbonaceous matter in a hydrothermal vein took a different analytical approach. Cavalazzi *et al*. [95] made an *in situ* analysis of carbonaceous filaments in a low temperature chert vein from the 3.42 Gyr Kromberg Formation, Barberton Greenstone Belt, South Africa. They documented specifically Ni associated with the carbonaceous filament, the latter interpreted as a microbial fossil on the basis of its morphology and the presence of C, H, O, N and S. Methanogenic or methanotrophic metabolic pathways were suggested to explain the presence of Ni. The biogenetic interpretation is strong and the metabolic pathway suggested is consistent with the subsurface environment in which the microorganisms lived. However, the presence of Ni could be also related to chelation of Ni from the surrounding fluids that would have been enriched in Ni (the vein cross-cuts ultramafic rocks).

**Figure 4.**
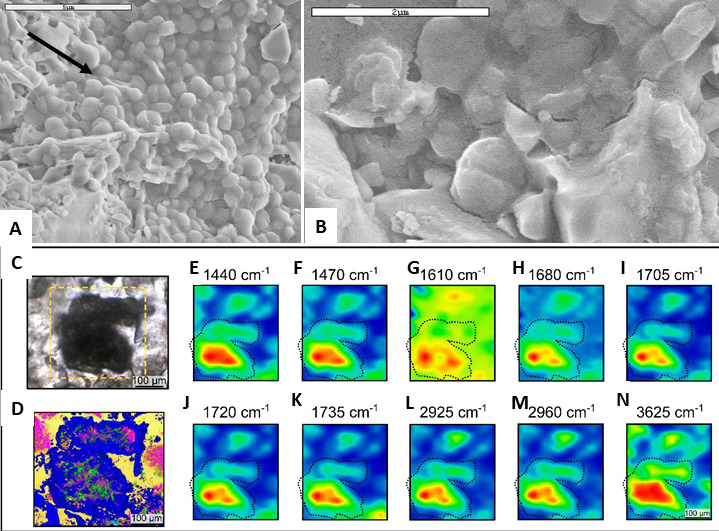
Purported chemotrophic microfossils from the Palaeoarchaean, from the 3.45 Gyr Kitty’s Gap Chert, Pilbara. (A) Electron micrographs of an interpreted colony of microorganisms on volcanic particles. (B) Detail of some of the preserved interpreted cells illustrating two sizes (with diameters averaging 0.8 and approx. 0.4 µm) suggestive of two species, as well as cell division. (Photomicrographs from Westall *et al.* [10]). (C,D) FTIR maps of the concentrations of different carbon and inorganic molecules associated with the carbonaceous matter (E, F = aromatic rings; G, H = Si O; I–K = carbonyl, carboxyl; J, K = aliphatic stretching; N = OH).

The latter study is rare in that the fossil remains of potential chemotrophs are seldom preserved. One comprehensive *in situ* investigation involved the study of interpreted colonies of chemolithotrophic microorganisms in littoral volcanic sands from the 3.45 Gyr Kitty’s Gap Formation in the Pilbara [96]. Westall *et al*. [9,16] had documented associations of small, carbonaceous, coccoidal structures (two size ranges, approx. 0.8 and approx. 0.4 µm diameter) embedded in a film-like substance that they interpreted as microbial in origin (figure 4A,B). The intimate association of the interpreted colonies with the surfaces of volcanic particles and volcanic dust, and their evident subsurface habitat, led Westall *et al.* to suggest that the structures might represent colonies of chemolithotrophic microorganisms.

In order to further examine the carbonaceous matter in the Kitty’s Gap sediments, Clodoré *et al*. [96] undertook a broad range of complementary analyses to investigate the elemental and molecular composition of the carbonaceous matter and its nanostructure (figure 4C,D). Relatively short and highly branched membrane lipids composed of five to eight alkane units that may represent archaeal membrane isoprenoid chains (cf. [97–99]) were interpreted from the CH_2_/CH_3_ ratios of the aliphatic in the carbonaceous matter. CH_3_/CH_2_ ratios, on the other hand, were consistent with both bacterial and archaeal origins [97,98], although the stronger contribution from archaeal membrane lipids may indicate an Archaea-dominated community. (It should be noted, however, that such ratios may not always to be applicable, at least not for the younger, 407 Myr Rhynie Chert biota [100]). This would place the existence of the last archaeal common ancestor (LACA) and the last bacterial common ancestor (LBCA) slightly earlier than shown in the chart of Moody *et al*. [13], i.e. closer to 3.5 Ga rather than closer to 3.3 Ga—at least in the Pilbara.

Clodoré *et al*. [96] also investigated the elemental composition of the carbonaceous matter in the Kitty’s Gap sediments. While transition elements, such as Ni, were enriched and could be interpreted as evidence of methanogenesis (cf. [95,101]), it is equally likely to be related to the concentration of ambient metals onto degrading organic matter during diagenesis. Nevertheless, the presence of degrading organic matter capable of chelating environmental transition metals is suggestive of former microbial colonies.

Similar enrichments of Ni associated with stellate carbonaceous clots formed *in situ* in fine, dust-like volcanic sediments of the 3.33 Gyr Josefsdal Chert in South Africa were also interpreted as indicating a possible methanogenic pathway [99], although here also the chelation of transition metals from the environment onto degrading organic matter is a likely explanation.

#### (ii) Phototrophy

**Figure 5.**
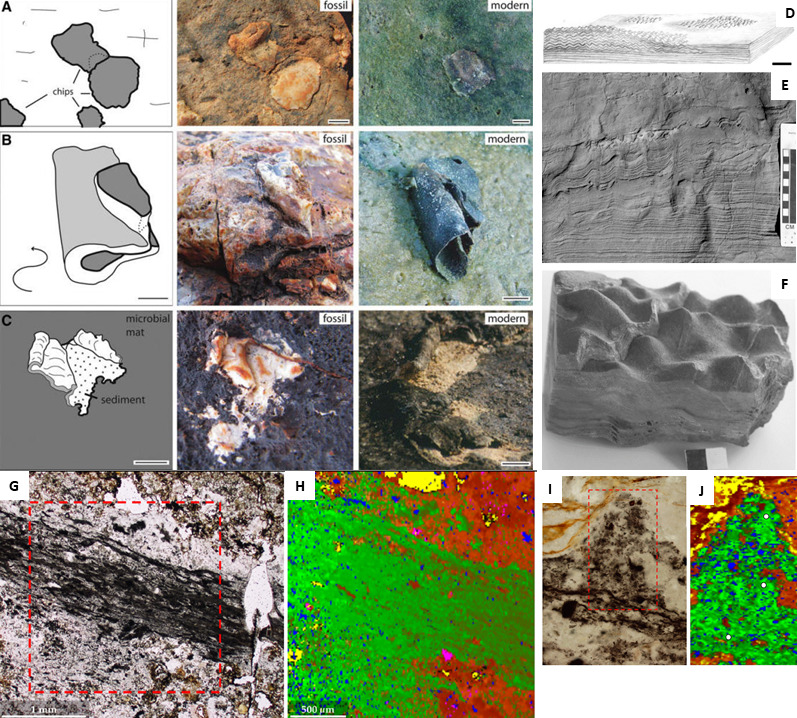
Palaeoarchaean photrophic microbial mats. (A–C) Various morphological aspects of MISS from the 3.48 Gyr Dresser Formation, Pilbara [11]. (D–F) Conical stromatolites from the 3.4 Gyr Strelley Pool Chert, Pilbara [7]. (G–J) Tabular and tufted phototrophic biofilms from the 3.47 Gyr Middle Marker Chert, Barberton [103] (G,I: optical; H,J: Raman spectral image with carbon in green and quartz in red).

There are many occurrences of phototrophic biofilm and mats in the form of MISS [102] and stromatolites in the Palaeoarchaean [20] (figure 5). Evidence for phototrophic organisms can be found in the oldest Palaeoarchaean rocks, from the 3.48 Gyr Dresser Formation in the Pilbara [11,91–93] and the 3.47 Gyr Middle Marker Chert in Barberton [103], and they continue to appear, with increasing importance, throughout the Archaean and into the Proterozoic, when they reach their apogee [20]. Considering the importance of photosynthesis for further development of life, the electronic supplementary material contains a brief overview of the origins of photosynthesis and its expression in sediments and in the rock record.

The oldest known stromatolites occur in the 3.48 Gyr Dresser Formation, Pilbara [91–93]. The Dresser Formation also hosts MISS, formed by phototrophic mats in an environment interpreted to be similar to a modern sabkha (figure 5A-C; [11]). While considering the possibility that the phototrophic microorganisms constructing the MISS were cyanobacteria (the Palaeoarchaean environment was anoxic), Noffke *et al.* noted that the mats could have also been formed by anaerobic organisms using H_2_S instead of H_2_O as the electron donor, similar to modern *Chloroflexus* or sulfur- or iron-oxidizing bacteria, such as Beggiatoa.

The Dresser Formation also hosts evidence for subaerial phototrophic microbial mats formed in geyserites precipitated in hot spring pools/vents [92]. These include stromatolites exhibiting microbial palisade fabric and gas bubbles preserved in inferred mineralized, exopolymeric substance. In modern geyserites, gas bubbles in microbial extracellular polymeric substances are formed by oxygen expulsed during oxygenic photosynthesis. In this case, Djorkic *et al.* [92] suggest that the gas could be of either biogenic or hydrothermal origin.

Another example of interest is the 3.4 Gyr Strelley Pool Chert in which Hofmann *et al*. [6] and Allwood *et al*. [55,104–106] describe a range of different kinds of stromatolites formed in different kinds of environments controlled by variations in water depth, sediment influx and hydrothermal activity on a carbonate platform (figure 5D-F). The Strelley Pool Chert stromatolites are the oldest known stromatolites that formed on a sediment-starved, peritidal carbonate platform facing the open ocean. Interestingly, *in situ* carbon isotope measurements indicate the presence of microorganisms using a number of metabolic pathways, including the Calvin–Benson–Bassham cyles, as well as the Wood–Ljungdahl pathway, or methanogenesis/methanotrophy [107].

In the Barberton Greenstone Belt, interpreted phototrophic microbial biofilms and mats occur in the 3.47 Gyr Middle Marker (figure 5G–F; [103]), the 3.42 GyrBuck Reef Chert [108,109], the 3.33 Gyr Josefsdal Chert [10,71,110], and in the 3.2 Gyr Moodies Group sandstones [12,56,111,112]. The backdrop of these sediments is deposition in shallow water environments flanking emerged volcanic edifices. Hickman-Lewis *et al*. [113] documented geochemical evidence for significant riverine input, at least associated with microbial mat horizons, that they considered to indicate the presence of continents. However, the Palaeoarchaean rocks of the Barberton Greenstone Belt document generally low-lying exposed terrains with volcanoes but there is no evidence for significant continental exposure related to horizontal plate tectonics before 3.3 Ga [114].

All of the manifestations of phototrophy in the Palaeoarchean occurred in anaerobic environments, as far as can be determined, even on a microbial scale (cf. [115]). Nevertheless, as noted above, small amounts of oxygen would have been produced though direct photolysis of the water surface, as well as through the production of radicals, such as H_2_O_2_, and through photolysis of high pressure water vapour exiting shallow hydrothermal vents. Thus, distinguishing abiotic from ephemeral biogenic sources of oxygen will be very difficult.

### (e) Mesoarchaean and oxygenic photosynthesis

As in the Paleaoarchean, there is much evidence for phototrophy in the Mesoarchaean. However, in contrast to the Palaeoarchaean sedimentary successions, the Mesoarchaean sediments formed in environments influenced by continental erosion related to the development of horizontal plate tectonics. Exposure of the granitic cores of the continents and their subsequent erosion led to greater influx of nutrients, especially phosphorus, into the surrounding seas.

**Figure 6.**
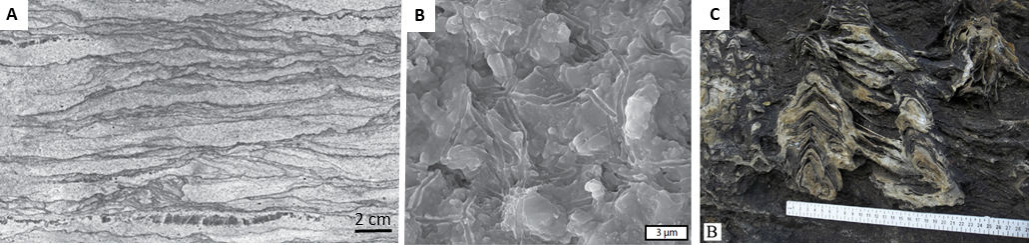
Diverse fossiliferous structures from the Palaeo-Mesoarchaean. (A,B) MISS and possible phototrophic microbial casts from the 3.2 Gyr Moodies Group coastal sediments, Barberton [12]. (C) Stromatolites from the 2.9−3.0 Gyr Chobeni Formation, Pongola Supergroup [118].

It is from morphological and geochemical signatures in these sediments that suggestions of possible oxygenic photosynthesis have been made. The 3.22 Gyr Moodies Group coastal and deltaic sediments contain MISS [12,56,111,112,116,117] and evidence of the earliest known forms of cavity-dwelling microbial communities [12] (figure 6A,B). The extent of the mat biomes, their similarity to modern cyanobacterial mats (e.g. macroscopic tufts, evidence for gas production and accumulation in bubbles and domes, and their widespread occurrence and presumably fast growth rate) and the large size of the cells from the finer-grained Moodies sediments [119], suggest to Homann *et al*. [112] and Homann [117] that they may have been formed by oxygenic phototrophs, the ancestors of cyanobacteria [112].

Certain geochemical proxies from the Moodies Group sediments have been interpreted as evidence for oxidative environmental conditions, such as Ce anomalies from a Moodies iron formation, although they most likely represent rare earth element mobility during more recent weathering [120]. In a review of the geochemical evidence for the rise of environmental oxygen associated with Moodies Group iron formations, Robbins *et al*. [70] also note proxies, such as high Mn/Fe ratios together with significant correlations of the latter and δ^56^Fe values [121]. Slightly younger sediments in the 3.2 Gyr Fig Tree Group exhibit uranium mobilization [122].

Indications of possible photosynthesis were interpreted from 2.9 Gyr sediments in the Chobeni Formation, Nsuze Group (Pongola Supergroup, South Africa) hosting stromatolites [118,123,124] (figure 6C) and MISS [102] formed in a siliciclastic, volcanoclastic carbonate and tide dominated shallow marine environment open to ocean. The presence of manganiferous shale and BIF in the Mozaan Group led Ossa [125] to hypothesize localized oxidizing conditions related to biogenic oxygenic photosynthesis that led to the oxidation of dissolved Mn^2+^ in the shallow marine environment. This interpretation followed on from an investigation of Mo isotopes by Planavsky *et al*. [126], who concluded that Mo isotope variability in the sediments was linked to the transport of Mo attached to Fe or Mn oxyhydroxides, with the Mn component transported under oxidizing conditions. Siahi *et al*. [124] suggest that a small, negative Ce anomaly associated with some of the samples from the stromatolitic carbonates may reflect ephemeral oxygenating conditions.

Further evidence for local oxygenated conditions during the deposition of the Pongola sediments comes from sulfur isotope measurements. Eickmann *et al*. [127] measured multiple sulfur and iron isotopes in early diagenetic pyrites, which exhibited consistently negative Δ^33^ S values in pyrite, suggesting photochemical reactions under anoxic atmospheric conditions. At the same time, large mass-dependent sulfur isotope anomalies of approximately 30‰ in δ^34^S were attributed to microbial sulfate reduction. The iron isotope values (δ^56^Fe) of −1.31 to −0.88‰ measured in the pyrites, were interpreted to indicate Fe oxidation in oxygen-bearing shallow oceans coupled with biogenic Fe reduction during diagenesis. This would suggest the existence of local Fe cycling in an oxygen oasis under a generally reduced Mesoarchaean atmosphere during Pongola times. For comparison, note the δ^56^Fe values of −1.2% to +1.2% that were measured in the 3.8 Gyr Eoarchaean BIF by Czaja *et al*. [60] and attributed to anoxygenic photoferrotrophic processes.

## 3. General considerations on oxygenic photosynthesis

The general understanding of the evolution of oxygenic photosynthesis is that it arose from anoxygenic photosynthesis. Cardona [17] summarized the reasons, thus: (i) the photochemical reaction centres used in anoxygenic photosynthesis are more primitive than those in oxygenic photosynthesis, (ii) the probability of acquiring photosynthesis via horizontal gene transfer is greater than the probability of losing photosynthesis, and (iii) there is no evidence for the existence of a transitional intermediate anoxygenic system utilising both type 1 and type II reaction centres. The conclusion is that the origin of anoxygenic photosynthesis pre-dates the origin of oxygenic photosynthesis.

Early anoxygenic phototrophy would have been limited by the number of available electron donors, i.e. H_2_ and Fe^2+^ [128]. Even if water was used as an electron donor, oxygenic photosynthesis would have been limited by the lack of availability of phosphorous and fixed nitrogen on the early Earth [129]. Thus, the early environments would have favoured anoxygenic phototrophs over oxygenic ones since the former could readily access H_2_ and Fe^2+^.

Investigations dealing with the evolution of oxygenic photosynthesis based on phylogenetic studies use different molecules, which result in different evolutionary trees depending upon the molecule used. For example, Xiong *et al*. [130] concentrated on (bacterio)chlorophyll biosynthesis genes from all photosynthetic lineages, which showed that purple bacteria occur at the base of all photosynthetic microorganisms and that heliobacteria are located at the base of oxygenic photosynthesizers. The molecular phylogeny for the *bch/chl* genes would argue strongly that cyanobacteria were late-evolving organisms.

On the other hand, more recent phylogenetic trees based on analysis of sequence and structural comparisons of specific components of photosynthesis, such as the reaction centres and proteins of photosystem II, and that use Bayesian-relaxed molecular clocks, suggest a very early rise in oxygenic photosynthesis at about 3.8 Ga (e.g. [129]) or between 3.5 and 3.2 Ga [19]. A large number of concomitant energy-consuming processes needed to have emerged in order to arrive at the water-splitting capability. They include a large increase in the structural complexity of photosystems I and II that went hand in hand with the increased complexity of the associated light-harvesting complexes. Thermodynamic coupling between both photosystems needed to be developed and important changes needed to be made to the entire electron transport chain in order to cope with oxidizing conditions. Finally, assembly and repair processes that could cope with the presence of oxygen needed to be developed. Given the high energetic costs of these processes, the first water-oxidizing reaction centres may have been active only for brief amounts of time in the absence of efficient repair [129]. The latter authors rationalize that ‘that such rates were only possible near the origin of reaction centres when life was still “figuring out” how to do photosynthesis for the first time.’ In a later article summarizing the data regarding molecular clocks suggesting that the core proteins of photosystem II (PSII) were involved in oxygenic photosynthesis, Sánchez-Baracaldo *et al*. [19] propose that oxygenic photosynthesis emerged in the Paleoarchean (3.6−3.2 Ga), while the last common ancestor of modern cyanobacteria emerged in the Mesoarchean (3.2−2.8 Ga).

Opposed to the molecular clock indications of an early evolution of oxygenic photosynthesis, Martin *et al*. [36] propose a scenario in which an early form of photosynthesis, photothiotrophy, would have been globally widespread as the predominant source of primary production in the photic zone of Archaean oceans, and was a trait of early oxygenic phototrophs. An ancestor of reaction centre 2 prior to the evolution of water oxidation would have been the cause of the whiffs of oxygen prior to the GOE (cf. [67]). A similar hypothesis was proposed previously by Nisbet & Fowler [131].

## 4. Conclusions

Life has been on Earth since the Hadean, although lack of a well-preserved rock record precludes dating its advent. The emergence of life, considered to be a natural evolution of chemistry to biology (cf. [1]), was conditioned by the anaerobic, ultramafic, geological environment of the early Earth.

However, the early environment was characterized by imported extraterrestrial materials and organics, organics formed by atmospheric processes, as well as organics formed and reworked by crustal processes, which complicates identification of biogenetic signatures in the Eo-Palaeoarchaean, in particular, as well as potential information regarding early microbial metabolisms. Nevertheless, the abundance of biosignatures as of the Palaeoarchaean, and as early as 3.48 Ga, suggests a diverse community including chemotrophs and phototrophs that lived possibly also in the Eoarchaean, despite the paucity of well-constrained biosignatures from that epoch [20]. Both Bacteria and Archaea groups were identified in rocks up to 3.48 Gyr old [96,113]. This pushes back the divergence of LBCA and LACA in the time tree chart of Moody *et al*. [13] (figure 1).

Apart from gross metabolic attributions, such as chemotrophy or phototrophy, the evidence for more refined metabolic strategies in the Eo-Palaeoarchaean is circumstantial and interpretive. With respect to the metabolic time chart suggested by Lyons *et al*. [37], there is no evidence of nitrogen fixation in the Eo-Palaeoarchaean. The localized oxygen oases suggested by a number of geochemical proxies that may have existed in Pongola Supergroup times (2.9−3.0 Ga) in the Mesoarchaean, with their stromatolites and MISS, may have supported nitrogen fixation [59]. Various sulfur metabolisms have been interpreted from diverse biosignatures in the Palaeoarchaean (e.g. [71,94,132]). Of the Fe-based metabolisms, photoferrotrophy may already have existed during Isua times (3.7−3.8 Ga), although it will be necessary to ascertain the lack of influence from abiotic signatures. Finally, methanogenesis has been invoked as a possible metabolic strategy for interpreted chemolithotrophic microorganisms from the 3.48 Gyr Dresser Formation chert veins [8,89], the 3.45 Gyr Kitty’s Gap sediments, the 3.42 Gyr Buck Reef Chert [95] and possibly the 3.33 Gyr Josefsdal Chert chemotrophs [99]. However, the carbon isotope signature of the first may have been influenced by crustal abiotic carbon, and the Ni signatures of the last three may also have been due to diagenetic enrichment processes.

The major question here is the emergence of oxygenic photosynthesis. Different molecular clocks propose different timings for the emergence of oxygenic photosynthesis, possibly during the Palaeoarchaean, but the emergence of cyanobacteria, as such, came much later. How photosynthesis arose and whether oxygenic photosynthesis developed through lateral gene transfer from anoxygenic photosynthesis, or emerged before or at the same time, is still debated. Further refinement in molecular clocks and phylogenetic trees may elucidate this aspect. The question here is, to what extent can the geological record baseline the different hypotheses?

There may be evidence of photosynthesis in 3.7−3.8 Gyr Isua rocks in the form of stromatolites and iron isotopes, if these interpretations are confirmed. The 3.48 Gyr Dresser Formation in the Pilbara and the 3.72 Gyr Middle Marker in Barberton both have evidence of photosynthesis in the form of stromatolites and MISS. The carbon isotope signatures are not diagnostic because of the combined influences of abiotic extraterrestrial and crustal processes, as well as the reworking of a phototrophic signature by heterotrophic microorganisms within microbial biofilms and mats. The measured signatures thus overlap with those of other metabolisms. Other morphological indications of photosynthesis in the form of biofilms and stromatolites are common in the Palaeoarchaean rocks from the Pilbara and the Barberton Greenstone Belt and, by the Mesoarchaean (as of 3.2 Ga), oxygenic photosynthesizers have been suggested to be the originators of MISS and fossils in the Moodies Group sandstones formed on land (rivers and deltas) and in the adjacent shallow sediments. However, geochemical indications of possible local environmental oxygen are only associated with the later Fig Tree Group BIFs [122] and 2.95 Gyr Pongola Supergroup sediments and stromatolites (e.g. [127,133,134]).

One of the main limiting factors for documenting the possibility emergence of oxygenic photosynthesis in the Palaeoarchaean is the problem of abiotic means for producing small amounts of oxygen. Phototrophic microbial mats necessarily grew in sunlit, shallow water environments. Planktonic phototrophs that were probably common in the Proterozoic and definitely important in the Neoproterozoic are not known from the Palaeoarchaean (the attribution of large, lenticular or spindle-shaped structures and acritarchs from the Palaeo-Mesoarchaean (e.g. [5,7,15,103,132,135,136]) is not yet understood; in any case, they were not common). The flux of UV radiation to the surface of the early Earth was much higher than today [38]. Thus, photolysis of water at the surface of the shallow water environments in which the phototrophic microbial mats lived would have produced free oxygen. Moreover, if there were shallow hydrothermal vents in the vicinity, these may also have produced free oxygen or reactive oxygen species, such as H2O2, through photolysis of high pressure water vapour exiting shallow vents. Disentangling the contributions of abiotic oxygen from potential contributions from biota will be arduous.

The earliest life forms were circumscribed by their geological habitats. Hydrothermal and volcanic activity strongly constrained chemotrophic life forms, while the phototrophs were opportunists, taking hold whenever there was a cessation in volcanic activity during the Palaeaoarchaean, becoming more widespread during the volcanically quieter, Mesoarchaean in coastal and subaerial, continental sandy environments.

## Data Availability

This is a review and all attributions to previous studies are correctly made. Supplementary material is available online [137].
